# The influence of various regions of the FOXP2 sequence on its structure and DNA-binding function

**DOI:** 10.1042/BSR20202128

**Published:** 2021-01-06

**Authors:** Monare Thulo, Megan A. Rabie, Naadira Pahad, Heather L. Donald, Ashleigh A. Blane, Cardon M. Perumal, J. Carlos Penedo, Sylvia Fanucchi

**Affiliations:** 1 Protein Structure-Function Research Unit, School of Molecular and Cell Biology, University of the Witwatersrand, Johannesburg 2050, South Africa; 2 Centre of Biophotonics, School of Physics and Astronomy, University of St Andrews, St Andrews KY16 9SS, U.K.; 3 Biomedical Science Research Complex (BSRC), School of Biology, University of St Andrews, St Andrews KY16 9ST, U.K.

**Keywords:** DNA binding, Forkhead domain, FOXP, Leucine zipper, oligomerisation, Transcriptional regulation

## Abstract

FOX proteins are a superfamily of transcription factors which share a DNA-binding domain referred to as the forkhead domain. Our focus is on the FOXP subfamily members, which are involved in language and cognition amongst other things. The FOXP proteins contain a conserved zinc finger and a leucine zipper motif in addition to the forkhead domain. The remainder of the sequence is predicted to be unstructured and includes an acidic C-terminal tail. In the present study, we aim to investigate how both the structured and unstructured regions of the sequence cooperate so as to enable FOXP proteins to perform their function. We do this by studying the effect of these regions on both oligomerisation and DNA binding. Structurally, the FOXP proteins appear to be comparatively globular with a high proportion of helical structure. The proteins multimerise via the leucine zipper, and the stability of the multimers is controlled by the unstructured interlinking sequence including the acid rich tail. FOXP2 is more compact than FOXP1, has a greater propensity to form higher order oligomers, and binds DNA with stronger affinity. We conclude that while the forkhead domain is necessary for DNA binding, the affinity of the binding event is attributable to the leucine zipper, and the unstructured regions play a significant role in the specificity of binding. The acid rich tail forms specific contacts with the forkhead domain which may influence oligomerisation and DNA binding, and therefore the acid rich tail may play an important regulatory role in FOXP transcription.

The FOX superfamily comprises one of the largest classes of transcription factors, consisting of over 100 members in species ranging from yeast to humans. Its members share a conserved DNA-binding domain of a winged helix fold, referred to as the forkhead domain (FHD) [1]. The FOX proteins are further subdivided into 19 subfamilies (FOXA-FOXS) based on sequence similarity both in the forkhead domain and in the remainder of their sequence [2]. FOX transcription factors are ubiquitously expressed during embryonic development as well as throughout adult life, and they play important developmental roles in numerous organs including the heart, lungs, liver, brain, and pancreas [2]. They are also involved in a range of diverse essential biological functions including regulation of the immune system, chromatin remodelling, glucose metabolism, ageing and longevity, and language and cognition [3]. Because of the significant role these transcription factors play in such a variety of tissues and cells, it is not surprising that they have been linked with numerous diseases including cancer [4].

The FOXP subfamily consists of four members (FOXP1–4). Unlike most other FOX transcription factors, members of the FOXP subfamily typically repress rather than activate transcription [5]. FOXP1, 2, and 4 regulate gene expression in the brain, lung, and gut [6–9]. FOXP2 is most well-known for its involvement in spoken language [6,7,10] and both FOXP1 and FOXP2 have been associated with cognitive disorders such as schizophrenia and autism [11,12]. FOXP3 is expressed in regulatory T cells where it plays a role in the immune system [13]. Structurally, the FOXP proteins are characterised by an N-terminal poly-glutamine tract, a zinc finger and a leucine zipper motif in addition to the forkhead DNA-binding domain which is located near the C-terminal end of the protein [5]. FOXP1, 2, and 4 also have a highly acidic region of approximately 70 amino acids at the C terminus, termed ‘the acid-rich tail’.

Like other FOX proteins, the FOXP proteins interact with DNA by insertion of the forkhead domain helix 3 into the DNA major groove [14–18]. This interaction demonstrates distinct specificity towards the DNA sequence [19]. Unlike other FOX proteins, however, the FOXP proteins have the ability to form domain swapped dimers via the forkhead domain [14–16]. Furthermore, FOXP proteins are also capable of multimerising via their leucine zipper motif [5,20]. This multimerisation event can be homomeric but can also be heteromeric and different members of the FOXP family have been shown to hetero-associate [5,21–23]. Indeed, unlike their FOX siblings which interact with DNA as monomers, it has been stated that it is necessary for FOXP proteins to dimerise via the leucine zipper in order to interact with DNA [5]. This statement, while perhaps true *in vivo* with the full-length protein, is not a requirement *in vitro* as the isolated forkhead domain has been shown to bind DNA in monomeric form [14,24].

It is clear that in order to fully understand the way FOXP proteins bind to DNA and regulate transcription, it is necessary to understand the role played by each of the domains in the sequence. Does multimerisation play a significant part in transcriptional regulation in this subfamily? How exactly does such a large superfamily of ubiquitously expressed proteins have such specific non-overlapping functions, and do all parts of the sequence have a unique role to play in ensuring that the proteins perform their specific functions? While there is some evidence already available to suggest putative roles for certain parts of the sequence (e.g., the polyglutamine tract and zinc finger motif are believed to be involved in protein–protein associations, the leucine zipper has been shown to cause homo and heterotypic multimerisation and the forkhead domain is responsible for DNA-binding specificity [5,19,25]), it is not known how these domains, along with the remaining undefined regions of the sequence including the acid rich tail, function together to support a cohesive mechanism of transcriptional regulation. The objective of the present study, therefore, is to provide more clarity on how the two oligomerisation interfaces, the leucine zipper and the forkhead domain, as well as the interlinking disordered sequence including the acid rich tail, co-operate so as to contribute to the structure and DNA binding of the FOXP proteins.

## Experimental methods

### Expression and purification

The present study focused on five FOXP constructs. They were: FOXP1 LZ-END and FOXP2 LZ-END which contained the full stretch of sequence from the leucine zipper to the C-terminal end of the protein in both FOXP1 (UniProt ID Q9H334) and FOXP2 (UniProt ID O15409) respectively; FOXP2 LZ-FHD which contained the stretch of FOXP2 sequence from the leucine zipper to the end of the forkhead domain; FOXP2 FHD which contained the isolated forkhead domain and FOXP2 FHD-END which contained FOXP2 sequence from the beginning of the forkhead domain to the end of the protein including the acidic C-terminal tail.

The coding sequences for all five constructs were each inserted into the multiple coding site of a pET-11A plasmid (Novagen, Germany) which is under the control of a T7 promotor (Genscript, U.S.A.). FOXP1 LZ-END, FOXP2 LZ-FHD, FOXP2 FHD-END and FOXP2 FHD were overexpressed at 20°C as previously described for the FHD [14,26] and FOXP2 LZ-END was expressed under similar conditions except at 37°C. The overexpressed proteins were purified using IMAC chromatography on a HiTrap™ nickel charged column (GE Healthcare, U.S.A.) as previously described for the FHD [24,26,27]. Briefly, this included passing the proteins through the column in binding buffer (20 mM Tris-HCl, pH 7.6, 500 mM NaCl, 50 mM imidazole) followed by a high salt and detergent wash (20 mM Tris-HCl, pH 7.6, 1.5 M NaCl, 50 mM imidazole supplemented with 1% Triton-X and 0.5% Tween® 20), followed by a mild imidazole wash (20 mM Tris-HCl, pH 7.6, 500 mM NaCl, 100 mM imidazole), followed by a one-step elution (20 mM Tris-HCl, pH 7.6, 500 mM NaCl, 500 mM imidazole). The proteins were stored in a buffer containing 10 mM HEPES, pH 7.5, 200 mM NaCl and 2 mM DTT.

### Structural characterisation

Secondary structural characterisation of all five FOXP constructs was performed at 20°C using far-UV circular dichroism (CD) spectropolarimetry with a JASCO J-1500 instrument (JASCO inc). Measurements were recorded on 10 μM protein in the wavelength range of 190−250 nm using a path length of 0.2 mm and were an average of three replicates. All samples were filtered to 0.2 µm so as to remove aggregates or dust particles that may interfere with the polarisation of light. Raw data were converted from mdeg to mean residue ellipticity ([θ]_MRE_). Data analysis was performed using the SELCON algorithm on the DichroWeb server [28], and the significance of the results was estimated using an unpaired Student’s *t*-test. Thermal unfolding of the variants was monitored with far-UV circular dichroism over a temperature range of 20–80°C with a gradient of 1°C/min by following the signal at 222 nm – a wavelength that gives an indication of the α-helical content of the protein.

Fluorescence spectra were measured in triplicate at 20°C using a JASCO FP-6300 spectrofluorometer (JASCO inc). All protein samples at a concentration of 2–5 µM were excited at 295 nm and the emission spectra were recorded from 295 to 450 nm. Fluorescence emission intensity of each spectrum was then normalised for comparison.

Size exclusion chromatography (SEC) was used to investigate the native quaternary state of the constructs following incubation of 20 µM of each of the constructs at 20°C for at least 30 min. A Superdex® 200 10/300 GL column (GE Healthcare, U.S.A.) was used to size the longer proteins, FOXP1 LZ-END, FOXP2 LZ-END and FOXP2 LZ-FHD, and a HiLoad™ 16/60 Superdex® 75 prep grade column (GE healthcare, U.S.A.) was used for FOXP2 FHD and FOXP2 FHD-END. The columns were equilibrated with running buffer (500 mM NaCl, 10 mM HEPES, pH 7.5, 2 mM DTT) and calibrated using the Gel Filtration Markers Kit for Protein Molecular Weights 12–200 KDa (Sigma Aldrich, U.S.A.).

### Structural modelling

Of the five constructs, the only crystal structure available is that of the isolated FHD [14]. In order to obtain an idea of the structures of the remaining four constructs, FOXP1 LZ-END, FOXP2 LZ-END, FOXP2 LZ-FHD and FOXP2 FHD-END, structure modelling was performed using the I-TASSER (http://zhang.bioinformatics.ku.edu/I-TASSER) online server [29]. I-TASSER implements a hierarchical approach to modelling protein structures. Structural modelling through I-TASSER occurs in three steps: structural template identification, iterative structure assembly, and structure-based function annotation. The server identifies structural templates from the Protein Data Bank (PDB) by LOMETS and iteratively assembles the protein folds by threading input sequences to build the tertiary structure. The continuous fragments (> 5 residues) are excised from the LOMETS alignments and used to reassemble the structure by replica-exchange Monte Carlo simulations. The simulation trajectories are then clustered by SPICKER and are used as the starting state of the second round I-TASSER assembly simulation. Finally, the structures of the lowest energy are selected, which are then refined by a fragment-guided molecular dynamics procedure, with the purpose of optimising the hydrogen-bonding network and removing steric clashes [29].

In order to refine the models, energy minimisation was implemented to mitigate any unwanted, straining energy potential in the models that could cause unfavorable rotamer positions, bad angles and bonds, clashes and many other adverse conformational changes. The Schrödinger suite encompassing Maestro™ was used to facilitate the model refinement through several commands. A PDB model was imported into the Maestro™ workspace and subjected to refinement by the Protein Preparation Wizard. The following parameters were included under the import and process option: assignment of bond orders, addition of hydrogens, creation of zero-order bonds to metal, creation of disulfide bonds, deletion of waters beyond 5.00 Å from heterogen groups and the generation of heterogen states using Epik pH 7.00 ± 2. The run was preprocessed, reviewed and modified. Next, the model was optimised by removing water molecules, the convergence of heavy atoms to an RMSD of 0.3 Å and implementation of the OPLS3e force field. Subsequently, the MacroModel minimisation job was run. The models were further checked for bad bond angles and rotamers as well as Ramachandran outliers using MolProbity [30,31].

### DNA binding

#### Oligonucleotide

All DNA-binding studies in this work used a duplex cognate DNA sequence containing a single binding site (underlined): 5′-TTAGGTGTTTACTTTCATAG-3′. This particular DNA sequence has been shown to have a strong affinity for the FOXP2 FHD [19,24,32]. The oligonucleotide was synthesised as a duplex by Integrated DNA Technology, South Africa.

#### Electrophoretic mobility shift assay

In order to confirm that all constructs were folded in their correct, native 3D form and hence were functionally viable, electrophoretic mobility shift assays (EMSAs) were conducted on all constructs. In these assays, protein was mixed with DNA, resolved on an acrylamide gel and stained for DNA with SYBR® Gold Nucleic Acid Stain (Invitrogen, U.S.A.). In a typical EMSA, free DNA, due to its smaller size and greater electrophoretic mobility, is readily separated from DNA that is associated with protein. In this way DNA-binding can be identified via electrophoretic shifts where bands representing DNA complexed with protein will migrate slower through the gel relative to free DNA [33]. Prior to electrophoresis, 500 nM DNA oligonucleotide was mixed at increasing protein concentration (0–40 µM) in a binding buffer containing 10 mM HEPES, pH 7.5, 1 mM MgCl_2_, 100 mM KCl, 10% (w/v) glycerol and 0.1 mg/ml BSA. The samples were then incubated on ice for 30 min to allow for DNA–protein complex formation. To minimise streaky bands on EMSAs, the poly-acrylamide gels were subjected to an electrophoresis pre-run using a pre-run buffer (25 mM Tris, 192 mM glycine, pH 8.3) for 1 h at 100 V at 4°C. Following this the samples were loaded onto 10% continuous EMSA gels (10% (w/v) acrylamide, 0.69% (w/v) bis-acrylamide) and subjected to electrophoresis at 4°C for 2–4 h, using the pre-run buffer.

#### Fluorescence anisotropy

Fluorescence anisotropy was used to obtain further information about the binding interaction between each construct and DNA. When fluorophores are excited with polarised light, the degree of polarisation upon emission (anisotropy) is related to the size of the complex. Larger protein–DNA complexes with slower tumbling times will display greater anisotropy than free DNA. Fluorescence anisotropy experiments were conducted on a PerkinElmer LS-50B fluorescence spectrophotometer fitted with an anisotropy filter. Briefly, 500 nM ROX-5′-labelled DNA was incubated with increasing concentrations (0–6000 nM) of each of the five FOXP constructs in binding buffer (10 mM HEPES pH 7.5, 100 mM NaCl and 0.02% NaN_3_). Fluorescence anisotropy was measured at an emission wavelength of 605 nm following excitation with a wavelength of 580 nm. Data were obtained in triplicate and averaged. Errors are the standard deviation of the averaged replicates. The data were fit to a single site binding model using SigmaPlot version 13.0, from Systat Software, Inc., San Jose California U.S.A., www.systatsoftware.com. The significance of observed differences in dissociation constant (*K*_D_) for each of the constructs upon DNA binding was assessed using an unpaired Student’s *t*-test.

## Results

While a number of inferences have been made as to the purpose of the various FOXP domains through studies conducted both *in vivo* [5,6] and *in vitro* [25], the significance of the large proportion of undefined interlinking sequence in these proteins has not been considered. Bearing in mind that the undefined sequence constitutes well over 50% of the protein sequence, it is highly probable that these regions of the sequence cooperate with the defined domains so as to enable the protein to perform its function. Therefore, in the present study, our focus is specifically on the two dimerisation interfaces, namely the leucine zipper and the forkhead domain, as well as the interlinking sequence including the acid rich tail, and their contributions to structure, multimerisation and DNA binding of the FOXP proteins.

**Figure 1:**
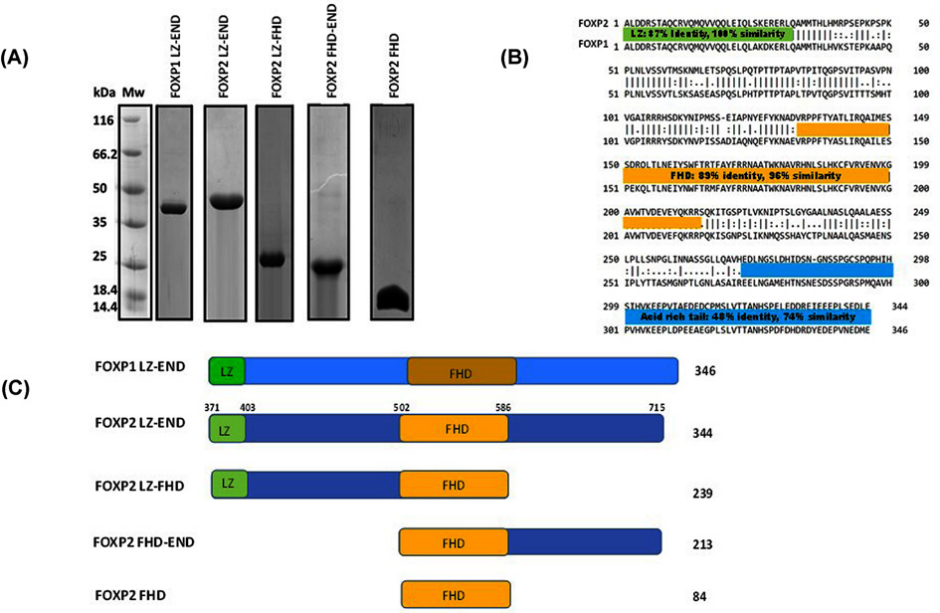
FOXP constructs used in the study (**A**) SDS-PAGE gel of each construct used in the present study following purification. Each construct is pure and migrates to its correct approximate predicted size. Predicted monomeric size of the constructs is: FOXP1 LZ-END ∼40 kDa, FOXP2 LZ-END ∼40 kDa, FOXP2 LZ-FHD ∼29 kDa, FOXP2 FHD-END ∼26 kDa, FOXP2 FHD ∼13 kDa. (**B**) Pairwise sequence alignment of FOXP1 LZ-END and FOXP2 LZ-END. The constructs share 68.5% identity and 85.3% similarity; however, this is not spread evenly throughout the sequence with the LZ and FHD sharing more similarity and the interlinking sequence and acid rich tail being less similar between the two sequences. The alignment was performed using EMBOSS Needle [34]. (**C**) A schematic showing the position of the domains on the sequence of the five FOXP constructs used in the present study. The number of amino acids in each construct is given on the right. FOXP2 numbering of the full-length protein is shown above the FOXP2 LZ-END species.

In order to address these matters, five different constructs were used in the present study (Figure 1C). All five constructs were successfully purified and migrated to their correct anticipated monomeric sizes on an SDS PAGE gel (Figure 1A). A number of different comparative studies were performed on the five constructs, including structural, oligomerisation and DNA binding studies. From these, we were able to make detailed conclusions about the specific roles played by the different parts of the protein sequence. Because the sequences of FOXP1 LZ-END and FOXP2 LZ-END are 100% similar in their leucine zipper regions and 96% similar in their forkhead domains (Figure 1B), a comparison between FOXP1 LZ-END and FOXP2 LZ-END will primarily give information on the acid rich tail and interlinking sequence as these regions share the least similarity and low identity between the two proteins. Comparing FOXP2 LZ-FHD with FOXP2 LZ-END will give information on the specific contribution of the acid rich tail, while comparing FOXP2 LZ-END with FOXP2 FHD-END will give information about the leucine zipper region. The FHD construct is useful for understanding how the forkhead domain behaves in isolation.

**Figure 2:**
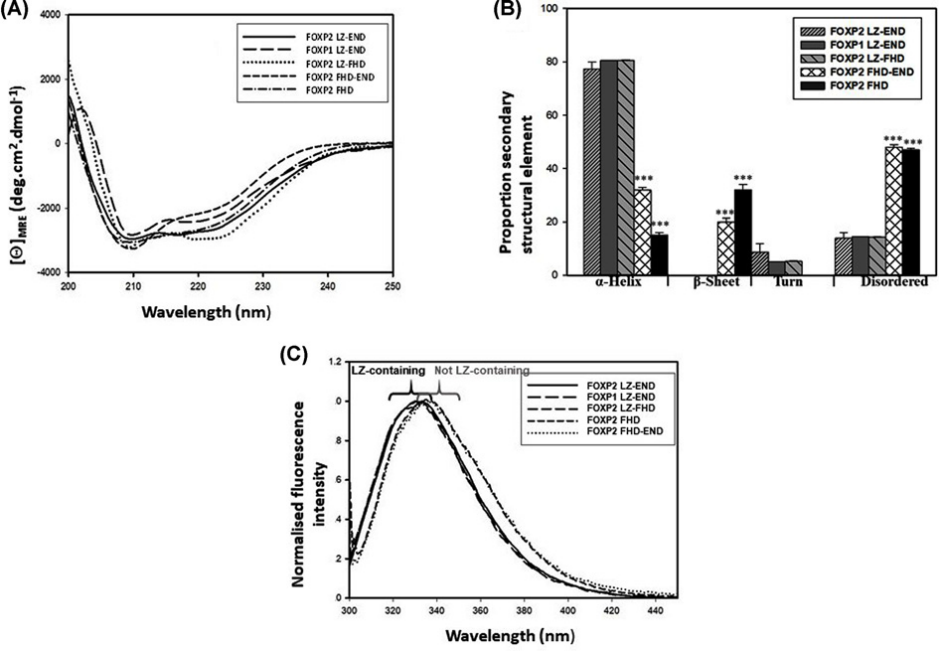
Secondary and tertiary structural characterisation of the five FOXP constructs (**A**) Secondary structural characterisation using far-UV circular dichroism. (**B**) Secondary structural content analysis of the circular dichroism spectra using DichroWeb [28]. The longer constructs have a higher proportion of helical structure but also more disorder. Statistical significance of the differences in proportion of secondary structural content was determined using an unpaired Student’s *t*-test (***P<0.001). (**C**) Normalised tryptophan fluorescence spectra (excitation wavelength 295 nm) of the five FOXP constructs. Maximum fluorescence intensity is observed at ∼330 nm for the longer, leucine-zipper-containing FOXP constructs and ∼336 nm for the forkhead domain variants.

**Figure 3:**
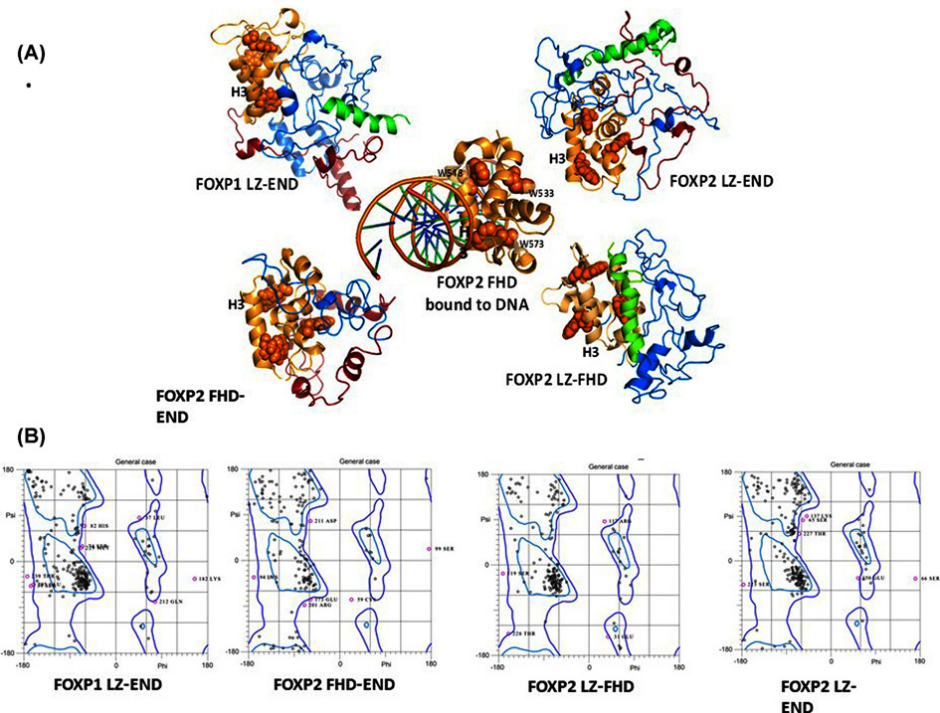
Molecular modelling of the FOXP constructs (**A**) Modelled structures of the constructs in comparison to the crystal structure of the FHD bound to DNA (PDB ID 2A07) [14]. The LZ region is green, the FHD is gold and the acid rich tail is red. The DNA-binding helix, H3, is shown in a similar orientation in each construct. The side chains of the three tryptophan residues are indicated as spheres. Models were created using I-TASSER online server [29,35]. Structures were generated in PyMol [36]. (**B**) Ramachandran plots for the *ab initio* models of each of the FOXP constructs. The models were generated and validated using a series of different software and programs, including I-TASSER online software [29,35], the Maestro suite in Schrödinger and MolProbity online tool [30,31]. Ramachandran plots were generated using MolProbity [30,31].

In order to interpret any structural contribution made by the different regions of the sequence, it was necessary to characterise each of the constructs in terms of their secondary structure (Figure 2A,B), their tertiary structure (Figure 2C) and their predicted 3D structure (Figure 3). All constructs displayed a circular dichroism spectrum characteristic of a predominantly alpha helical protein with a trough at 208 and 222 nm. Analysis of the spectra revealed that the three longer variants, which contain the leucine zipper, have a significantly higher proportion of helical content than the two shorter variants. This implies that the leucine zipper and the interlinking sequence between the leucine zipper and the forkhead domain are significantly more helical than the remaining portion of the protein. Because ∼80% of the longer constructs was shown to contain helical structure while only ∼34% of the sequence is predicted to form the structured regions of the leucine zipper and forkhead domain (Figure 1), the CD result implies that a large proportion of the connecting sequence in the FOXP structure is not disordered and indeed contains defined helical structure. The FHD and FHD-END constructs display a proportion of beta structure in addition to the alpha-helices and this is confirmed by the FHD crystal structure [14].

All five FOXP constructs contain three tryptophan residues and all three of these are located in the forkhead domain (Figure 3A). As with the CD spectra, the intrinsic tryptophan fluorescence spectra also indicate a difference between the three leucine zipper-containing constructs and the two smaller variants. There is a shift in the peak of the spectrum to a lower wavelength when in the presence of the leucine zipper, implying that in the leucine-zipper-containing constructs, the three forkhead domain tryptophan residues are in a more hydrophobic environment.

The 3D models of the constructs (Figure 3A) agree with the spectroscopic structural characterisations in that all the models appear predominantly helical with the FHD-END construct showing the least helicity as seen in the Ramachandran plot (Figure 3B) which is consistent with the CD data. The leucine zipper presents as a helix in all the models and the forkhead domain resembles that of the crystal structure. Although the quality of the models is not as high as would be expected for a crystal structure, the fact that they agree with experimental data and the fact that the MolProbity validation scores are within acceptable limits [31] (MolProbity scores ranging from the 50th percentile to the 65th percentile and MolProbity clash scores all being above the 97th percentile), means we can use the models to make plausible qualitative interpretations of the structure of the constructs as a supplement to the experimental data.

Interestingly, despite the large proportion of undefined sequence, the 3D model structures of the FOXP constructs all appear to be more globular than extended, with clear pockets of secondary structure evident in the interlinking regions which corresponds with the CD data (Figure 2B). The C-terminal acid rich tail in particular folds back to form specific contacts with the forkhead domain (Figure 3A).

While these predicted structures are informative, it is important to bear in mind that they represent monomeric models only and since the FOXP subfamily is believed to multimerise, via both the leucine zipper [5,37] and the forkhead domain [14,24], the way the protein behaves will likely be influenced by its multimeric structure.

**Figure 4:**
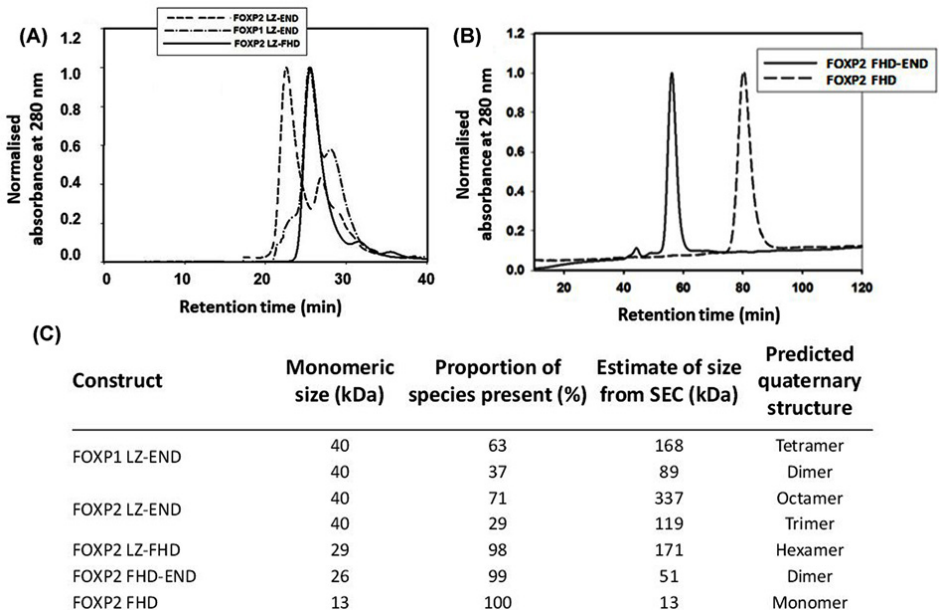
Determination of size and heterogeneity of the constructs (**A**) Gel filtration elution profiles of FOXP1 LZ-END, FOXP2 LZ-END and FOXP2 LZ-FHD. Size exclusion chromatography was performed on a HiLoad Superdex 200 Increase 10/300 GL column. Both FOXP1 and FOXP2 LZ-END constructs eluted as two peaks while FOXP2 LZ-FHD eluted as a single peak. (**B**) Gel filtration of the smaller FOXP2 FHD-END and FOXP2 FHD constructs was performed on a HiLoad 16/60 Superdex 75 prep grade column. Both these constructs eluted as a single peak. (**C**) Table showing the predicted size and predicted quaternary structure of each of the species separated by SEC.

Therefore in order to assess the contribution of the various parts of the sequence to the quaternary structure of the FOXP proteins, we performed size exclusion chromatography on the native protein constructs (Figure 4). This was performed using two separate columns of different range due to the difference in size of the constructs under study. Indeed, the results imply that four of the five constructs exist as multimers in solution with only the isolated forkhead domain eluting from the size exclusion column at its predicted monomeric size (Figure 4). Furthermore, and as reported by others [25], the constructs existed as mixtures of species of different sizes, particularly in the case of FOXP1 LZ-END and FOXP2 LZ-END, where two distinct peaks are seen in the elution profile (Figure 4A) despite a single pure band being detected on the SDS-PAGE gel (Figure 1A).

Predictions based on the monomeric size indicate that ∼70% of the FOXP2 LZ-END species could exist in oligomeric structures as large as octamers and ∼60% of the FOXP1 LZ-END species could exist as tetramers. Due to the large proportion of disordered sequence predicted in these structures, it is plausible that they do not fold into entirely globular structures and therefore the size exclusion results could give an over estimate of their true multimeric sizes due to their larger hydrodynamic volume. However, the 3D models do predict that even the disordered regions take on some structure with the models appearing to be mostly globular (Figure 3A) and so even if the exact sizes cannot be accurately determined, the SEC results clearly do point towards all four constructs exhibiting some degree of oligomerisation and for the purpose of this study, this qualitative result is sufficient. Oligomerisation via the leucine zipper in the three longer constructs also explains the fluorescence results, where the forkhead domain tryptophan residues appear to be in a more hydrophobic environment in the three leucine-zipper-containing constructs compared with the two shorter constructs (Figure 2C). This result suggests that multimerisation via the leucine zipper domain probably results in partial burial of the forkhead domain.

**Figure 5:**
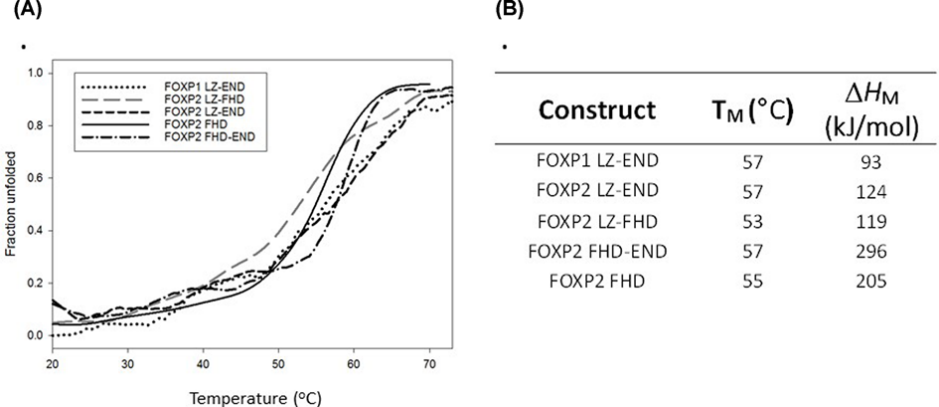
Thermal stability (**A**) Thermal melting curves of the FOXP constructs obtained by monitoring the far-UV CD ellipticity at 222 nm over a temperature range of 20–80°C. Thermal unfolding data were fitted using the global curve fitting wizard in SigmaPlot from Systat Software, Inc., San Jose California U.S.A. (**B**) Table showing the *T*_M_ for each of the constructs.

Thermal melting curves give an indication of protein stability, although Δ*G*(H_2_O) cannot be accurately obtained from these melting curves since they do not display reversibility [38]. The thermal melts of the five constructs (Figure 5) indicate similar *T*_M_ values but display differences in cooperativity which can be attributed to how homologous the solution is.

**Figure 6:**
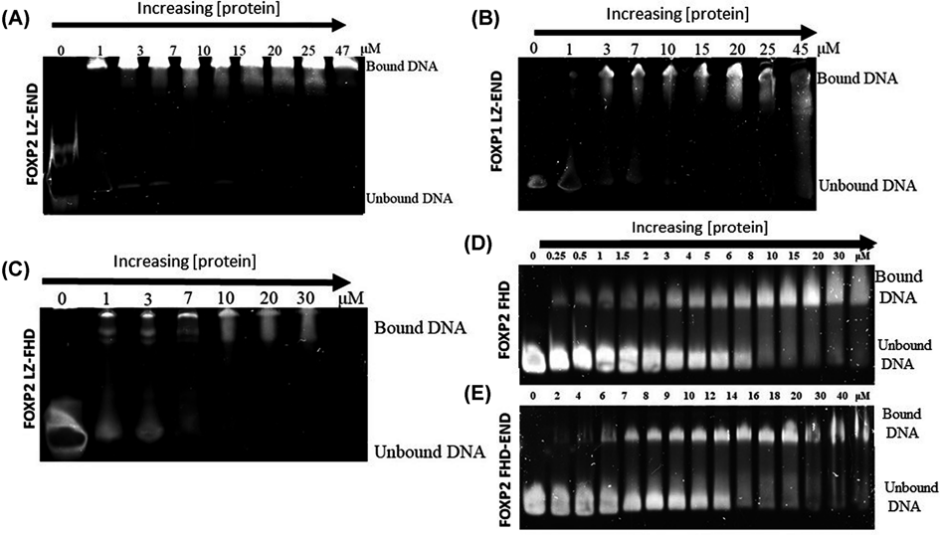
Protein−DNA complex formation of the FOXP variants as determined by electrophoretic mobility shift assay About 500 nM cognate DNA was incubated with increasing concentrations (5−40 μM) of: (**A**) FOXP2 LZ-END, (**B**) FOXP1 LZ-END, (**C**) FOXP2 LZ-FHD, (**D**) FOXP2 FHD, (**E**) FOXP2 FHD-END. All constructs showed DNA binding capability, suggesting that the FHD (DNA-binding domain) is correctly folded and accessible to DNA in all cases.

In addition to studying the structure of the various constructs, it was also necessary to study their DNA-binding behaviour. In order to assess whether each of the constructs was able to interact with DNA, electrophoretic mobility shift assays (EMSAs) were used (Figure 6). The EMSAs indicate that each of the constructs is capable of binding DNA. From this qualitative assessment, it appears that the three leucine zipper-containing constructs have the greatest affinity for the DNA although the leucine zipper is not a requirement for DNA binding. The gels also suggest that whereas the two smaller constructs appear to form a single complex with the DNA, the three leucine zipper-containing constructs may bind to the DNA as more than one species with varying affinity for the DNA. The slight smearing observed on the gels indicates that within the gel environment, the proteins do not remain associated with the DNA but repeatedly come off and on the DNA as they migrate through the gel [33].

**Figure 7:**
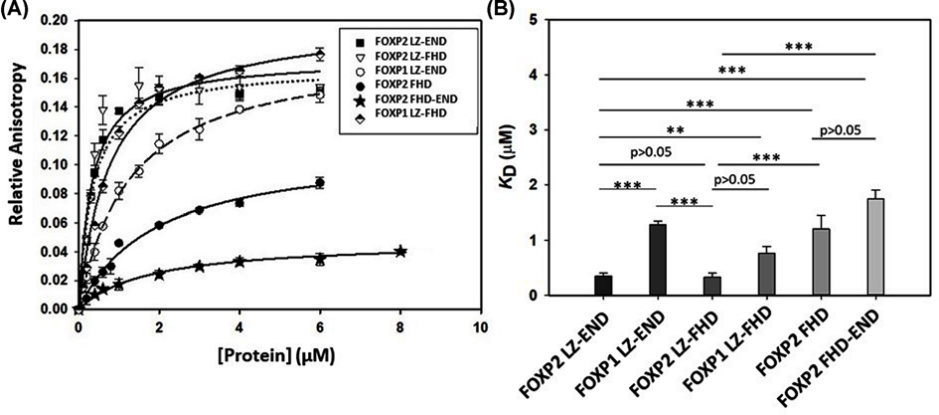
DNA-binding affinity studies (**A**) DNA-binding curves for the FOXP1 and FOXP2 constructs as determined by fluorescence anisotropy. For each experiment, 500 nM dsDNA containing a single FOXP2 binding site labelled at the 5′ end with ROX was incubated with increasing concentrations of each protein construct (0–6 μM). The fluorescence anisotropy at each concentration was measured and the data fitted to a single site binding model. (**B**) The statistical significance of the differences between the *K*_D_ of DNA binding of each variant was determined using a Student’s *t*-test (*P<0.05; **P<0.01; ***P<0.001). Errors indicate the standard deviation of three averaged replicates. The *K*_D_ obtained for DNA binding of FOXP1 LZ-END, FOXP2 FHD and FOXP2 FHD-END are: 1281.2 ± 70.4 nM, 1745.7 ± 15.9 nM and 1904.0 ± 25.1 nM, respectively which is significantly higher than 766 ± 12.1 nM, 347.1 ± 55.4 nM and 326.2 ± 73.2 nM for FOXP1 LZ-FHD, FOXP2 LZ-FHD and FOXP2 LZ-END, respectively.

Since all constructs were found to be capable of binding DNA, fluorescence anisotropy was used to compare the DNA-binding affinities of each of the constructs (Figure 7). The results confirm the EMSA predictions in that all the constructs showed a binding curve and hence interacted with the DNA. Furthermore, and as suggested by the EMSA, a significant difference in DNA binding affinity was detected between the leucine-zipper-containing FOXP2 constructs and the two shorter constructs. The leucine-zipper-containing FOXP2 constructs appear to interact with the DNA significantly more tightly than the shorter FOXP2 species that do not contain the leucine zipper. Interestingly, however, the leucine-zipper-containing FOXP1 LZ-END construct does not interact with the FOXP2 cognate sequence with as high an affinity as the FOXP2 LZ-END construct despite containing 100% similarity in the leucine zipper domain and 96% similarity in the forkhead domain. This implies that the interlinking sequence may play a significant part in affecting the affinity of the protein for DNA. In order to address this observation further, we used anisotropy to assess the DNA binding of a 6th construct – FOXP1 LZ-FHD. This construct is missing the acid rich tail. Interestingly, there is no significant difference in DNA-binding affinity between FOXP2 LZ-FHD and FOXP1 LZ-FHD and therefore the significantly weaker binding of FOXP1 LZ-END must be ascribed to the acid rich tail which is absent in the FOXP1 LZ-FHD construct.

## Discussion

The purpose of the present study was to investigate how the structure of FOXP proteins influences their DNA-binding function. A deep understanding of this concept will lead to a better appreciation of how this protein subfamily regulates gene expression. In order to answer this question, the region of the FOXP sequence extending from the leucine zipper to the C terminus was dissected into five constructs containing combinations of the individual structured domains as well as regions of disorder. Through these constructs, the manner in which the different parts of the sequence cooperate so as to produce functional protein was investigated. The stretch of FOXP sequence under study contains two oligomerisation interfaces namely the leucine zipper and the forkhead domain. The remainder of the sequence is thought to be disordered and has not been given much consideration in the literature to date, despite the fact that it constitutes over half the protein (Figure 1).

Structural studies indicate that all constructs take on a significant proportion of helical structure and it is clear from both the CD spectra (Figure 2A) and the 3D models (Figure 3A) that this helical structure is not only present in the predicted structured domains, but makes up a portion of the ‘unstructured’ region as well. The protein appears not to take on the classical ‘beads on a string’, extended structure but rather appears to be folded and globular, and even though regions of disorder do exist, they constitute under 20% of the backbone fold of the three larger constructs (FOXP1 LZ-END, FOXP2 LZ-END and FOXP2 LZ-FHD) and aren't represented at all in in the backbone structure of the two smaller constructs (FOXP2 FHD and FOXP2 FHD-END) (Figure 2).

The size exclusion chromatography results show that four of the five constructs are not monomeric and form higher order oligomers. Indeed, multimerisation via the leucine zipper is likely to be a property of all FOXP proteins as has been identified by others in FOXP3 [5,20] and FOXP2 [25]. Despite the fact that all five constructs have the potential to form multimers, they do not appear to do so with the same propensity. Our results show that FOXP2 LZ-END forms the largest complexes, with species potentially as large as octamers being detected, while the size of FOXP1 LZ-END multimers appears to be only about half of that (Figure 4A). Because the leucine zipper is 100% similar in these two constructs, this implies that it is not the leucine zipper alone but also the unstructured sequence including the acid rich tail that contributes to multimerisation. The only one of the five constructs that appears to be monomeric in the present study is the FHD. There is evidence that the isolated FHD can form a domain swapped dimer [14], but this was observed at the very high concentrations required for crystallisation and so its physiological relevance remains uncertain. Our work shows that the isolated forkhead domain exists as a monomer in solution at physiological concentrations and furthermore that it can bind DNA as a monomer [24]. Therefore, it is unlikely that the multimerisation events that we observe with the other four constructs are controlled by the forkhead domain. We can conclude that multimerisation occurs via the leucine zipper but is also influenced by the unstructured regions of the protein including the acid rich tail. This is further justified by the fact that the acid rich tail-containing FHD-END construct was shown to dimerise while the FHD alone is monomeric. Furthermore, multimerisation via the leucine zipper causes the environment of the forkhead domain to become more hydrophobic (Figures 2 and 4). Multimerisation thus has a direct effect on the position of the tryptophan-containing DNA-binding domain which implies that the formation of higher order oligomers could be linked to the DNA-binding function of the FOXP proteins. The EMSA results indicate that all the oligomeric species in the heterologous solution are capable of binding DNA (Figure 6). However this is merely a qualitative assessment and it is not clear from the EMSA if each species interacts with DNA with the same affinity. There is a possibility that the degree of oligomerisation can affect the way the protein interacts with DNA and since the degree of oligomerisation is also influenced by the unstructured regions [39], the unstructured regions will also have an effect on DNA binding. In general FOXP1 is less prone to form oligomers than FOXP2 and comparisons of the 3D modelled structures (Figure 3) tend to suggest that FOXP1 is less compact than FOXP2. Therefore, the less compact the FOXP structure is, the lower its propensity for higher order oligomer formation via primarily the leucine zipper, and the weaker its affinity for FOXP2 cognate DNA.

All five constructs used in the present study were shown to bind DNA (Figures 6 and 7). This is not too surprising because all five constructs contain the forkhead domain which has been demonstrated to be essential for DNA binding [25]. What is interesting, however, is that the isolated FHD construct does not show a strong affinity for the DNA. The FOXP2 leucine zipper-containing constructs show significantly greater affinity (Figure 7), implying that the leucine zipper is not only critical for multimerisation but plays a role in DNA binding as well. However, the strong affinity for the DNA cannot be ascribed to the leucine zipper alone because FOXP1 LZ-END, which shares 100% similarity in the leucine zipper region with FOXP2 LZ-END has a significantly lower affinity for the FOXP2 cognate sequence (Figure 7). This suggests that the acid rich tail and unstructured interlinking sequences assist with selectivity of binding and it is these regions of the sequence that are critical in ensuring that different members of the family display specificity towards different DNA sequences. Indeed, the binding affinity of an additional construct, FOXP1 LZ-FHD, supports our conclusion that the acid rich tail plays a role in binding specificity. In the absence of the acid rich tail, both FOXP1 LZ-FHD and FOXP2 LZ-FHD show similar binding affinities (*P* value = 0.1 therefore there is no significant difference between the *K*_D_ values). However, when the acid rich tail is included, FOXP1 LZ-END shows significantly lower affinity for DNA than FOXP2 LZ-END (Figure 7).

It is, therefore, possible that the acid-rich tail may actually play a significant regulatory role in DNA binding. According to the models, the acid-rich tail folds back and makes specific interactions with the forkhead domain (specific glutamate residues from the acid rich tail interact with specific arginine residues from the forkhead domain). This interaction could interfere with both oligomerisation and DNA binding, potentially allowing H3 to be better or worse exposed to the major groove. Therefore the possibility exists that the acid rich tail plays a finely-tuned regulatory role by affecting both multimerisation and DNA binding of the transcription factor. Indeed, this is not the only example of a flexible C-terminal acidic tail being involved in oligomerisation [40,41] or DNA interactions [42].

## Conclusion

The FOXP domains as well as the unstructured interlinking sequence have precise significant roles to play in the function of these proteins, specifically in terms of oligomerisation and DNA binding. FOXP2 appears to be more compact in its monomeric form than FOXP1 and this improves its propensity for higher oligomer formation. The multimerisation event is driven by the leucine zipper domain and in turn causes the DNA-binding forkhead domain to become partially buried in a hydrophobic environment. Hence, multimerisation also affects DNA binding. The oligomerisation of the FOXP proteins is not homologous and a number of species of varying oligomeric size can form. Each of these species is capable of binding DNA, however they may not all interact identically with the DNA so that heterologous multimerisation may be a means of regulation employed by these proteins. The multimerisation is also, in part, affected by the unstructured regions of the protein. Indeed, the flexible acid rich tail folds back and forms specific contacts with the forkhead domain through which it can influence DNA binding and possibly dimerisation via the forkhead domain. While the forkhead domain is essential for DNA binding, the affinity of this binding interaction is governed by the leucine zipper and the specificity of the association is under the control of the acid rich tail. Therefore the undefined, less conserved regions of the sequence play a part in ensuring that each member of the family is specific for its binding sequence by influencing the way the protein folds and multimerises. Our results suggest that more consideration should be given to the unstructured regions of the sequence in future, when considering regulation of transcription by these proteins.

## Accession Numbers

FOXP1 (UniProt ID Q9H334) and FOXP2 (UniProt ID O15409).

## Data Availability

All data are available from the corresponding author by request.

## Abbreviations

CD: circular dichroism
FHD: forkhead domain
FOX: forkhead box
LZ: leucine zipper
SEC: size exclusion chromatography
TF: transcription factor
